# The effect of object size on the sensitivity of single photon emission computed tomography: comparison of two CZT cardiac cameras and an Anger scintillation camera

**DOI:** 10.1186/s40658-014-0097-5

**Published:** 2014-12-31

**Authors:** Elazar A Bienenstock, Marguerite Ennis

**Affiliations:** Department of Nuclear Medicine, Etobicoke General Hospital of William Osler Health System, 101 Humber College Blvd., Markham, M9V 1R8ON Canada; Scarborough Cardiac Diagnostic Centre, 2391 Eglinton Ave E, Toronto, M1K 2M5ON Canada; Applied Statistician, 9227 Kennedy Rd, Markham, L3R 6H9ON Canada

**Keywords:** CZT camera, Coronary artery disease, Myocardial perfusion imaging, High-speed SPECT, Heart size

## Abstract

**Background:**

Heart sizes vary greatly across the spectrum of patients referred for myocardial perfusion imaging. We therefore performed a phantom study to explore under controlled circumstances how count rates change when different volumes containing the same amount of activity are scanned. Two dedicated cadmium-zinc-telluride cameras, the D-SPECT (Spectrum Dynamics, Caesarea, Israel) and Discovery 530c (D530c, GE Healthcare, Haifa, Israel), and the conventional SPECT Anger (A-SPECT, GE Healthcare, Haifa, Israel) camera are included in the study.

**Methods:**

Different heart sizes were represented by syringes of various column heights mimicking a range of cardiac diameters. Syringes with fixed activity were scanned at five different volumes by successively adding non-radioactive water to the syringes. This procedure was repeated five times on each of the three cameras. Raw count rates were recorded for each scan to determine whether count rates changed with syringe column height.

**Results:**

Using mixed-effect regression modeling, a linear relationship was found between count rate and water column height. For the D-SPECT, D530c, and A-SPECT, the changes in count rate for each centimeter increase in water column height were −1.75, +0.28, and −0.022 kilocounts per min per MBq, respectively (95% confidence intervals −1.89 to −1.61, 0.19 to 0.36, and −0.035 to −0.009); all effects are significantly different from each other and significantly different from zero. Average coefficients of variation were 0.080, 0.028, and 0.009.

**Conclusions:**

The D-SPECT demonstrated a significant progressive increase in count rate related to decreasing size of the imaged object. D530c count rate increased slightly with *increasing* column height. The Anger SPECT showed minimally increased count rates with decreasing column height, an order of magnitude smaller than the D-SPECT based on their relative coefficients of variation.

**Electronic supplementary material:**

The online version of this article (doi:10.1186/s40658-014-0097-5) contains supplementary material, which is available to authorized users.

## Background

Coronary artery disease is a leading cause of morbidity and mortality, particularly in advanced countries [1],[2] Myocardial perfusion scans are a key tool for the diagnosis and risk assessment of coronary artery disease. The expansion of imaging technology and interest in early disease detection have led to an estimated 9.1 million tests being performed annually in the United States in 2008 [3],[4]. The Anger scintillation gamma-camera design has been utilized since 1957 [5]. Recently two direct-conversion cadmium-zinc-telluride (CZT) detector cameras have become available: the D-SPECT (Spectrum Dynamics, Caesarea Israel) and Discovery 530c (D530c, GE Healthcare, Haifa, Israel). These have several advantages over scintillation gamma-cameras, including much faster scans [6].

Heart sizes vary greatly across the spectrum of patients referred for myocardial perfusion imaging. One standard deviation for myocardial mass in normal adult males ranges from 92 to 190 g [7]. The population undergoing myocardial perfusion scans includes normal women, which extends this range downward. The scanned population also includes a significant number of obese, diabetic, hypertensive, and ischemic patients at risk for left ventricular (LV) dilation, which extends the range upwards.

The development and enthusiastic acceptance of ultrasensitive cardiac CZT cameras highlights the importance of scan speed in contemporary nuclear medicine practice. Higher scan speed increases patient comfort, convenience, and throughput; reduces motion artifact; or facilitates radiotracer dose reduction. Recently, the scan speed of these CZT cameras was compared [8].

It has been our experience that when using the automated myocardial count level imaging function of the D-SPECT, stress scans are occasionally completed in less than 1 min rather than the average 2 min when using rest and stress doses of approximately 333 and 999 MBq, respectively. Paradoxically, these short duration scans demonstrated images of excellent quality. The fact that these short acquisition times tended to occur in small patients raised the question of a possible relationship between cardiac size and myocardial count rate. To investigate this, we performed a phantom study using a fixed amount of activity spread over increasing volumes to determine the effect of object size alone on count rate. We included the D-SPECT, D530c, and the conventional Anger SPECT (A-SPECT) camera in the study because, while it is known that these three cameras have different sensitivities [8], their response to changes in object size has not been compared. Based on a review of the camera mechanisms, we hypothesize that only for the D-SPECT, count rate would increase significantly with decreasing heart size.

## Methods

### Instrumentation

This study involved the use of a the D-SPECT camera at the Scarborough Cardiac Diagnostic Center (SCDC) and a D530c camera at Etobicoke General Hospital (EGH). The Anger SPECT (A-SPECT) camera used in this study was a Millennium VG (GE Healthcare, Haifa, Israel) at EGH. All were in Toronto, ON, Canada. The dose calibrator from SCDC was transported to the EGH to calibrate the dose calibrators to each other by measuring various activities in both devices in rapid succession. The D-SPECT and D530c both use identical square CZT modules. Each module consists of a 16 × 16 pixelated CZT crystal backed by a proprietary application-specific integrated circuit. The D-SPECT employs nine rotating detector columns (DCs) in an L-shaped array that translates between rotations to view spaces between detectors. Each rectangular DC consists of four vertically stacked CZT modules behind a very-high-sensitivity parallel-hole tungsten collimator. Each square collimator hole corresponds to one pixel etched into the CZT crystal surface. The D-SPECT requires a brief pre-scan following which the technologist draws a region of interest (ROI) to define the LV for cardio-centric DC movements [9]. Utilizing count-rate information from the pre-scan, the D-SPECT adjusts scan time in 1-s increments to attain a predetermined number of counts. The D530c employs an L-shaped array of 19 stationary pinhole units arranged in three rows. Each unit consists of a tungsten-tipped lead-bodied pinhole collimator with an effective aperture of 5.1 mm, projecting onto four CZT modules arranged in a 2 × 2 square [10].

### Preparation of syringe sets and data acquisition

**Figure 1:**
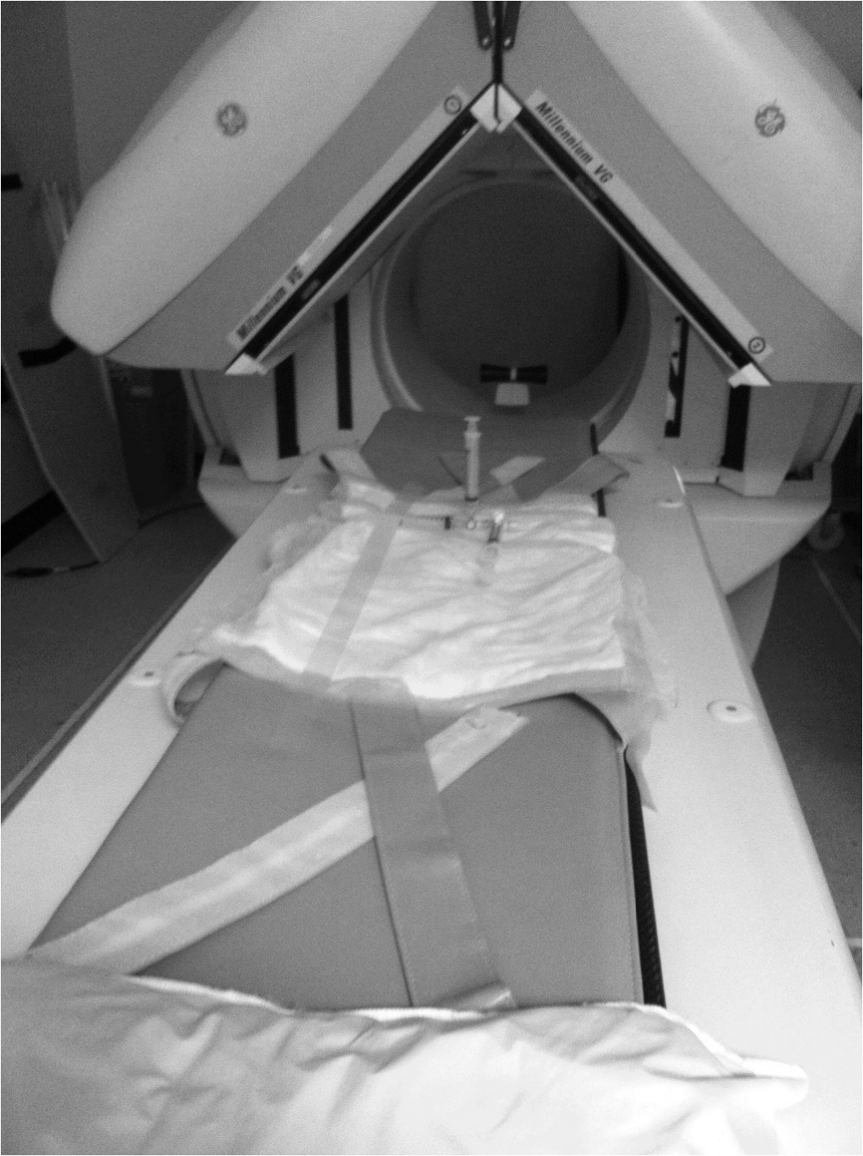
**Experimental setup.** Syringe-set at 1 cc in the A-SPECT camera just before rotating the detector to the start position.

The ready and reproducible production of different sized objects with intrinsically equal activity was achieved by repeatedly adding non-radioactive water to successively lengthen the activity column of a syringe containing Tc-99 m as follows. Two syringes were attached to a three-way stopcock at right angles paralleling both arms of the L-shaped CZT detectors and the Anger camera detectors in L mode at the start position. The setup at the EGH included 10-mL (B to D) syringes measuring 82 mm from the stopcock hinge to the 10-mL mark. At SCDC, 12-mL (Terumo, Shibuya, Japan) syringes were used measuring 78 mm from the stopcock hinge to the 10-mL mark (Figure 1). A solution of 14 to 46 MBq of Tc-99m in less than 1 cc of water was prepared and approximately half was injected into each of the two empty syringes. Water was added to both syringes through the 3rd stopcock port, topping-up both syringes to 1 cc. The horizontal syringe was taped to the imaging table with the plunger head pointing towards the vertical detector arm. The vertical syringe’s plunger required pruning to avoid collision with the upper CZT detector arms as the syringe is filled with water. Following the scan, 2 cc of water was added to each syringe. The time of dose calibration and start of each scan were recorded for decay correction. Without moving the syringe set, this process was repeated until five unique paired-column-height measurements of the same activity were obtained for each syringe set. Five syringe sets were scanned on each of the three cameras for a total of 75 measurements (5 column heights × 5 syringe-sets × 3 cameras). The column height measurements were 3.4 to 7.5 cm for the D-SPECT and 3.0 to 7.8 cm for the D530c and A-SPECT, due to the fact that the dimensions of the syringes and stopcocks supplied at SCDC were slightly different from those at the EGH. The arbitrary syringe set activities overlap the clinically encountered myocardial activity range of 8 to 24 MBq, based on 0.6% to 1.8% myocardial uptake [11] of 1,332 MBq Tetrofosmin-Tc-99m. Visual inspection of the images confirmed homogeneous activity throughout the extended water columns after each infusion of non-radioactive water, indicating good mixing during water injection. The entire experiment was conducted in the absence of external attenuation, eliminating potential confounding effects of variable external attenuation.

Scan duration was 2 min on both CZT cameras and 10 min and 13 s on the A-SPECT. A 2-min duration matches average D-SPECT clinical stress scan time and has been suggested for the D530c [6]. Ten minutes is at the lower limit of conventional A-SPECT stress scan duration. Shorter scan times would underestimate A-SPECT and D-SPECT count rates by increasing the proportion of lag time due to detector motion. The D530c’s fixed detectors suffer no lag time.

#### D-SPECT syringe imaging

Superimposed on the D-SPECT's pre-scan is a vendor-rendered circle centered 5.8 cm from the anterior camera surface and 5.2 cm from the lateral surface, indicating preferred heart position. For each series of measurements, the syringe set was positioned with the stopcock hinge at the center of this circle and not moved until the series was completed. On each pre-scan a tight ROI was drawn around the activity columns of the syringe set in a standard manner to avoid variability due to the shape of the ROI.

#### D530c syringe imaging

Optimal heart position is indicated by vendor-rendered crosshairs at 12 and 15 cm from the anterior and lateral camera surfaces, respectively, in plane with the central row of pinholes. For each series of measurements, the syringe set was positioned such that the stopcock hinge was at the crosshairs and it was not moved until the series was completed.

#### A-SPECT syringe imaging

A central point was chosen on the imaging table, and the syringe set is positioned at this point for each series. The syringe set was not moved between scans of the same series. Scans were performed with 30 stops (60 projections) for 18 s. Rotation diameter was 18 cm.

### Count quantification

Raw projection counts purely summed from all detectors were compared. Because all activity was from the syringe-sets, reconstruction to exclude background activity was unnecessary. As the D-SPECT scan progresses, the growing count number is refreshed on the acquisition monitor. The final value after scan completion was used. GE software was used to sum counts from the D530c’s 19 pinholes and from A-SPECT’s 60 projections.

The data for each camera consisted of 5 series, each series consisting of the count rates corresponding to 5 different water column heights. The measured counts were normalized to an activity of 1 MBq by using the dose calibrator activity measurement for each series, decay-corrected to the time of the scans. Counts were further divided by the scan duration to obtain counts per minute per megabecquerel.

### Statistical analysis

The coefficient of variation (CV) of the count rates was calculated for each series and the hypotheses that the CV's were the same for all three cameras tested using ANOVA. Regression modeling was used to obtain estimates of the count rate vs. object size relationship, in the form of a mixed model that accounted for the fact that observations were not completely independent but are correlated within the series. We fitted different intercepts and slopes for the three cameras and used random intercept effects for the series. Variances were allowed to differ by camera. The model was fitted by maximum likelihood and parameterized in two ways: one to test whether the slopes of the three cameras were significantly different from each other (using interactions) and the other to test whether the slopes differed significantly from zero. Residual analyses were satisfactory. All analyses were performed using S-PLUS 6.2 (Insightful Corp, Seattle, USA).

## Results

**Figure 2:**
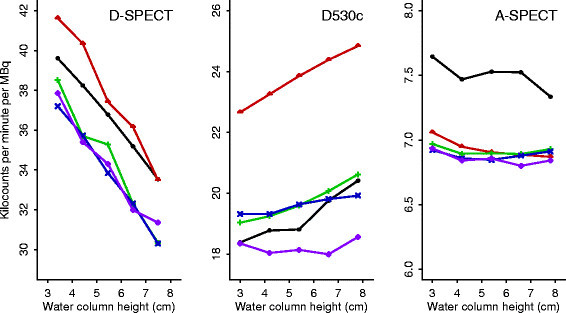
**Count rates as a function of syringe water column height.** A series of measurements at five different syringe heights was obtained by successively adding non-radioactive water to the syringe to lengthen the activity column. Five series are shown for each of the three cameras D-SPECT, D530c, and A-SPECT.

The relationship between count rates and the water column height is shown in Figure 2 with data in Table 1. The estimated slopes for the D-SPECT, D530c, and A-SPECT respectively from the mixed-effects model were −1.75, 0.28, and −0.022 kilocounts per min per MBq for each centimeter increase in water column height (95% confidence intervals −1.89 to −1.61, 0.19 to 0.36, and −0.035 to −0.009, respectively); these slopes were all significantly different from each other (data not shown) and significantly different from zero (Table 2). Thus, D-SPECT and A-SPECT's count rates increased as the water volume decreased while D530c showed the opposite pattern. The average CV's were 0.080, 0.028, and 0.009, respectively (*P* < 0.0001 for the null hypothesis that the CV for the three cameras were equal, Table 1). The CV's show that, while both D-SPECT and A-SPECT had negative slopes, the D-SPECT slope was much steeper relative to the average sensitivity of the camera than the A-SPECT slope (average sensitivities for the D-SPECT, D530c, and A-SPECT were 35.4, 20.1, and 7.02 kilocounts per min per MBq, respectively).

**Table 1:** **Count rates corresponding to different syringe water column heights**

D-SPECT	Water column height	3.4 cm	4.4 cm	5.5 cm	6.5 cm	7.5 cm		
		Kilocounts per minute per megabecquerel					CV^a^	Mean CV^b^
	Series 1	39.62	38.25	36.79	35.19	33.54	0.066	0.080
	Series 2	41.63	40.35	37.43	36.16	33.49	0.086	
	Series 3	38.53	35.70	35.28	32.30	30.33	0.092	
	Series 4	37.20	35.73	33.84	32.31	30.30	0.081	
	Series 5	37.86	35.39	34.30	31.98	31.35	0.077	
D530c	Water column height	3 cm	4.2 cm	5.4 cm	6.6 cm	7.8 cm		
		Kilocounts per minute per megabecquerel					CV^a^	Mean CV^b^
	Series 1	18.38	18.78	18.81	19.76	20.42	0.043	0.028
	Series 2	22.66	23.26	23.86	24.39	24.84	0.037	
	Series 3	19.03	19.25	19.60	20.07	20.62	0.033	
	Series 4	19.32	19.32	19.63	19.81	19.92	0.014	
	Series 5	18.35	18.04	18.14	18.00	18.57	0.013	
A-SPECT	Water column height	3 cm	4.2 cm	5.4 cm	6.6 cm	7.8 cm		
		Kilocounts per minute per megabecquerel					CV^a^	Mean CV^b^
	Series 1	7.65	7.47	7.53	7.52	7.33	0.015	0.009
	Series 2	7.06	6.95	6.91	6.89	6.87	0.011	
	Series 3	6.97	6.89	6.90	6.89	6.93	0.005	
	Series 4	6.92	6.86	6.85	6.88	6.91	0.005	
	Series 5	6.94	6.84	6.86	6.80	6.84	0.007	

**Table 2:** **Modeled relationship between count rate and water column height**

Fixed effects	Coefficient value	Standard error	Degrees of freedom	***P*** -value ^a^
Intercept	44.917	0.776	57	<.0001
D530c vs. D-SPECT	−26.300	1.049	12	<.0001
A-SPECT vs. D-SPECT	−37.779	1.021	12	<.0001
Slope for the D-SPECT	−1.747	0.072	57	<.0001
Slope for the D530c	0.277	0.043	57	<.0001
Slope for the A-SPECT	−0.022	0.007	57	0.0016
**Random effects**	**Standard deviation**	**Standard deviation adjustment per camera**		
Intercept	1.42	D-SPECT	D530c	A-SPECT
Residual	0.497	1	0.706	0.108

A mixed-effect regression model was used to estimate the relationship between count rate and water column height for each camera. The model fitted different fixed-effects intercepts and slopes for the three cameras and used random intercept effects for the series, with heteroscedasticity by camera.

^a^From *t*-test for testing that the coefficient equals zero.

## Discussion

The D-SPECT demonstrated significantly improved count rates with decreasing activity volume, a feature not previously encountered in nuclear medicine cameras. This camera feature is explained by the D-SPECT’s internal mechanics. According to Spectrum Dynamics (personal communication), each of the D-SPECT's nine DCs initially acquires images at 120 steps across a 110° arc, with equal time spent at each position. After the pre-scan, the computer adjusts DC movement so that 60% to 70% of angles view the LV as defined by the technologist-rendered ROI and 30% to 40% view the remainder of the chest. We can describe the 60% to 70% left ventricular views as the central portion of the field of view (CPFOV). As the heart size decreases, the DC angular intervals in the CPFOV become finer and more closely stacked causing increased overlap of consecutive DC images, at the expense of slightly coarser peripheral sampling. Thus, the virtual detector contracts or expands the CPFOV in response to heart size. Denser oversampling of smaller hearts increases cardiac count rate. The increase in sampling density and count rate with CPFOV shrinkage resembles ‘thickening’ of the detector as it contracts, like a muscle or rubber band. Another analogy that may capture this effect is a searchlight gliding over a field. A smaller search area enjoys improved illumination because the searchlight dwells longer at each location.

In contrast to the D-SPECT, fixed field of view cameras (D530c and A-SPECT) did not demonstrate similarly improved count rates with decreased column height. In fact, D530c count-rate increased slightly with *increased* column height. Further study may help assess whether this occurred because more of the activity was perpendicular to the pinholes with increased column height. The A-SPECT's count rates showed a small but statistically significant increase with decreasing column height. The increase is unlikely to be important clinically and is likely due to decreased self-attenuation by the smaller column. Although decreased self-attenuation may also increase D-SPECT count rates, the small scale of this effect cannot account for the much larger increase in count rate observed for the D-SPECT with decreased column height. It should be noted that, because these cameras have different resolution and efficiency characteristics, count rate does not reflect the resulting contrast to noise ratios that can be achieved for a given acquisition time.

Beyond the D-SPECT's adaptable virtual detector, there are supplemental causes for the rapid and high quality scans of small hearts that originally lead to this investigation. Images of smaller hearts are less noisy due to a higher count density, since total LV counts are shared by fewer milliliters or voxels. Additionally, small hearts are associated with smaller patients [12]. Decreased soft tissue attenuation in small patients allows more photons to escape the body and reduces scattered photons that deliver incorrectly assigned points of origin. This leads to higher count rates and better resolution. Voxel count density and attenuation affect all camera systems equally and unrelated to the D-SPECT’s increased count rate in small hearts identified in our phantom study.

CZT cameras possess small fields of view that are quite sensitive to heart position [13]. Smaller patients' hearts are more ideally positioned in the field of view. Additionally, closer proximity to the detector should improve image resolution and count rate for both cameras. Because only CZT cameras scan in direct chest contact, the improved heart-detector proximity due to decreased chest size in smaller patients is proportionately much more significant for CZT than A-SPECT cameras. For the D-SPECT, the center of the circle likely allows all nine DCs to view the heart from the closest average distance. Although D-SPECT uses a parallel hole collimator and Anger camera parallel hole collimator efficiency does not change with distance, D-SPECT sensitivity should decrease with increased distance from the heart. This is because of the size difference between A-SPECT and D-SPECT collimators. The Anger cameras have large detectors where the sensitivity lost per individual collimator-hole is offset by an equal increase in the number of collimator holes viewing an object as it recedes. Since the D-SPECT's 4-cm wide DCs do not span the average internal end systolic LV diameter [14], the number of collimator holes exposed to the heart does not increase in step with the decrease in individual collimator hole sensitivity as the object recedes. For the D530c, the crosshairs location presumably minimizes average myocardial displacement from the 19 pinhole apertures, since pinhole sensitivity falls with increasing angle and distance [15].

From a resource management perspective, extra high quality scans are ‘overexposed’ and present an opportunity to decrease radiopharmaceutical dose and/or scan-time. Although all cameras register higher count rates in smaller patients, only the D-SPECT capitalizes on this by shortening scan duration, thanks to its scan strategy that targets LV counts rather than a predetermined exposure time. The D-SPECT scan times could potentially be shortened even further for smaller hearts by targeting count density rather than total counts. LV volume might be estimated from the pre-scan. BMI input could further refine count-targets because improved heart detector proximity and less scatter in smaller patients could allow scan completion with lower than normal count-density. Currently, the Spectrum Dynamics recommends outlining the pre-scan LV with generous margins. As this study indicates, a tighter LV margin will increase count-rate by concentrating the CPFOV. Further study may reveal if tighter ROIs can be drawn to increase scan speed without degrading image quality.

In locales where heart sizes are smaller due to ethnic variations [16], this may mitigate slower scan speed associated with D-SPECT versions employing fewer DCs.

Other cameras could potentially personalize scan time without adding a time-consuming pre-scan by measuring LV count rate during early acquisition, provided that the exposure time adjustments described above could be completed well before the scan would normally end. D530c is particularly well suited for this because its inherent full-time SPECT configuration permits scan termination at any instant.

### Limitations

While different sized cardiac phantoms would be ideal, they are not available. Initially we attempted to use different sized hollow spheres to approximate the size and shape of the left ventricle. However, small random variations in dose-calibrator sphere-activity assays marred count-rate/megabecquerel comparisons between the different sized spheres. Since the syringe set only required repositioning five times for each camera, and care was taken to place it at the same spot each time, variability due to source location was minimal. Location was undefined only for the A-SPECT, but is unlikely to be relevant. The D-SPECT ROI was allowed to vary with object size as it would in practice, but its shape was standardized to avoid differences in ROI between repeat scans.

## Conclusions

This study confirms a significant volume effect on count rate for the D-SPECT due to its mechanically adaptable virtual detector. This design feature provides increased scope for acquisition optimization and has not been previously encountered in nuclear medicine cameras.

Smaller hearts increase the D-SPECT's count rates due to its mechanics and higher count-rates increase the D-SPECT's scan speed due to its scan strategy. Therefore, for any scan-speed evaluation or comparison of the D-SPECT with other cameras, heart-size or object size must be taken into account.

Targeting count density rather than absolute LV counts may optimize D-SPECT scan speed for all patient sizes. BMI input may further reduce count targets and scan time by leveraging multifactorial improved spatial resolution in small patients. The D530c is well suited to switch from a time-based to a count-based scan strategy to also personalize scan times. Benefits of personalizing scan time include shorter average scan time and extended scan time when needed.

## Authors’ original submitted files for images

Below are the links to the authors’ original submitted files for images. Authors’ original file for figure 1 Authors’ original file for figure 2

## Abbreviations

A-SPECT: Anger SPECT
BMI: body mass index
CPFOV: central portion of the field of view
CV: coefficient of variation
CZT: cadmium-zinc-telluride
D530c: Discovery 530c
DC: detector column
EGH: Etobicoke General Hospital
LV: left ventricular
ROI: region of interest
SCDC: Scarborough Cardiac Diagnostic Center
