# Supplementary figures and images for: The effect of object size on the sensitivity of single photon emission computed tomography: comparison of two CZT cardiac cameras and an Anger scintillation camera
Source: EJNMMI Phys. 2014 Dec 31;1:97. doi: 10.1186/s40658-014-0097-5 (PMC4545452; doi:10.1186/s40658-014-0097-5)

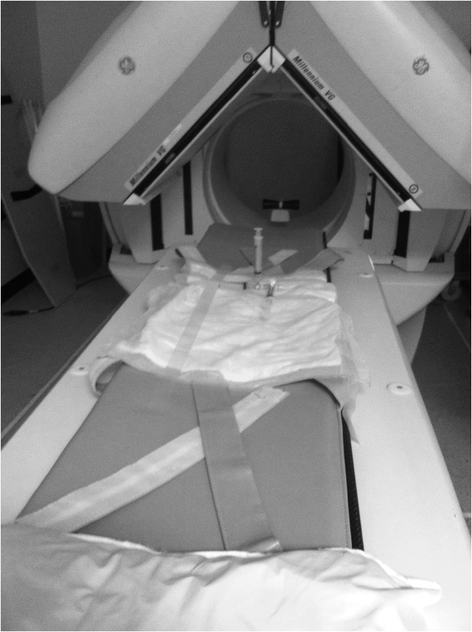

Supplement: Supplementary file 1 — Authors’ original file for figure 1 [file 40658_2014_97_MOESM1_ESM.gif]

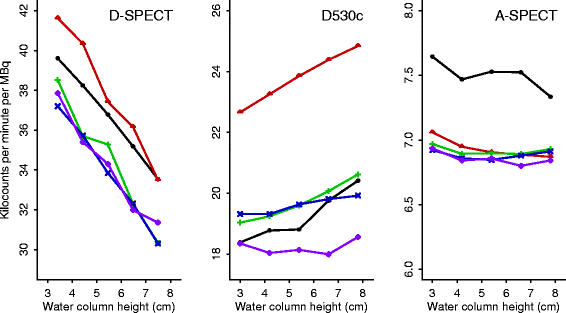

Supplement: Supplementary file 2 — Authors’ original file for figure 2 [file 40658_2014_97_MOESM2_ESM.gif]
